# Supplementary material for: Timing of whole genome duplication is associated with tumor-specific MHC-II depletion in serous ovarian cancer
Source: Nat Commun. 2024 Jul 18;15:6069. doi: 10.1038/s41467-024-50137-y (PMC11258338; doi:10.1038/s41467-024-50137-y)
Supplement: Supplementary file 1 — Supplementary information [file 41467_2024_50137_MOESM1_ESM.pdf]

Supplementary information file for:

Timing of whole genome duplication is associated with tumor-specific MHC-II depletion in serous ovarian cancer

Authors

Burdett, Nikki L[1,2,3], Willis, Madelynne O[1], Pandey, Ahwan[1], Twomey, Laura[1], Alaei, Sara [1,4], Australian Ovarian Cancer Study Group† [1], Bowtell, David DL[1,2], Christie, Elizabeth L[1,2]*

Affiliations

1. Peter MacCallum Cancer Centre, Melbourne, Victoria 3000, Australia
2. Sir Peter MacCallum Department of Oncology, The University of Melbourne, Victoria 3010, Australia
3. Box Hill Hospital, Eastern Health, Box Hill, Victoria, 3128, Australia
4. Australian Regenerative Medicine Institute, Monash University, Clayton, Victoria 3168, Australia

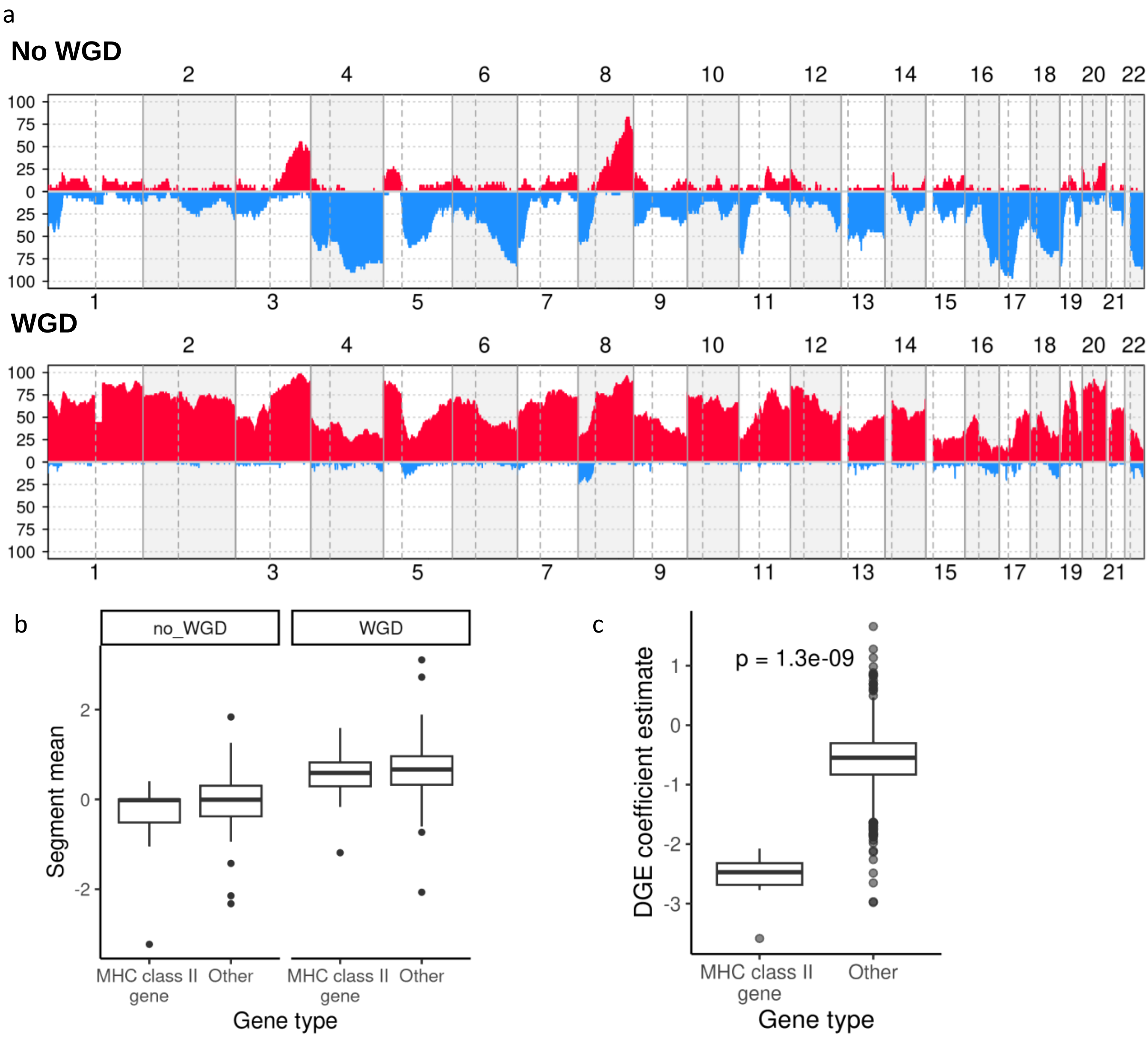

Supplementary Fig. 1. Whole genome duplication copy number and expression analyses.

a. Copy number frequency plots by chromosome for patients in the ICGC cohort with no whole genome duplication (WGD; top) and with WGD (bottom). Gain/loss threshold = ±0.4, gains colored red and losses colored blue. b. Boxplots of copy number segment means in cytobands 6p21.31 – 6p25.22 by whether gene is an MHC class II gene or any other gene; p = 0.09, glmm. n = 79 patient samples c. Boxplots of coefficient estimates from DGE model in cytobands 6p21.31 – 6p25.22 by whether gene is an MHC class II gene or any other gene; p <0.001, Wilcoxon's test. n = 79 patient samples. For figures b and c: Left and right whiskers terminate at the minimum and maximum values no further than 1.5× interquartile range; center line represents median (50th percentile); left and right boundaries of box represent the first (25th percentile) and third (75th percentile) quartiles, respectively; outlying values are plotted as individual points beyond whiskers. Source data are provided as a Source Data file.

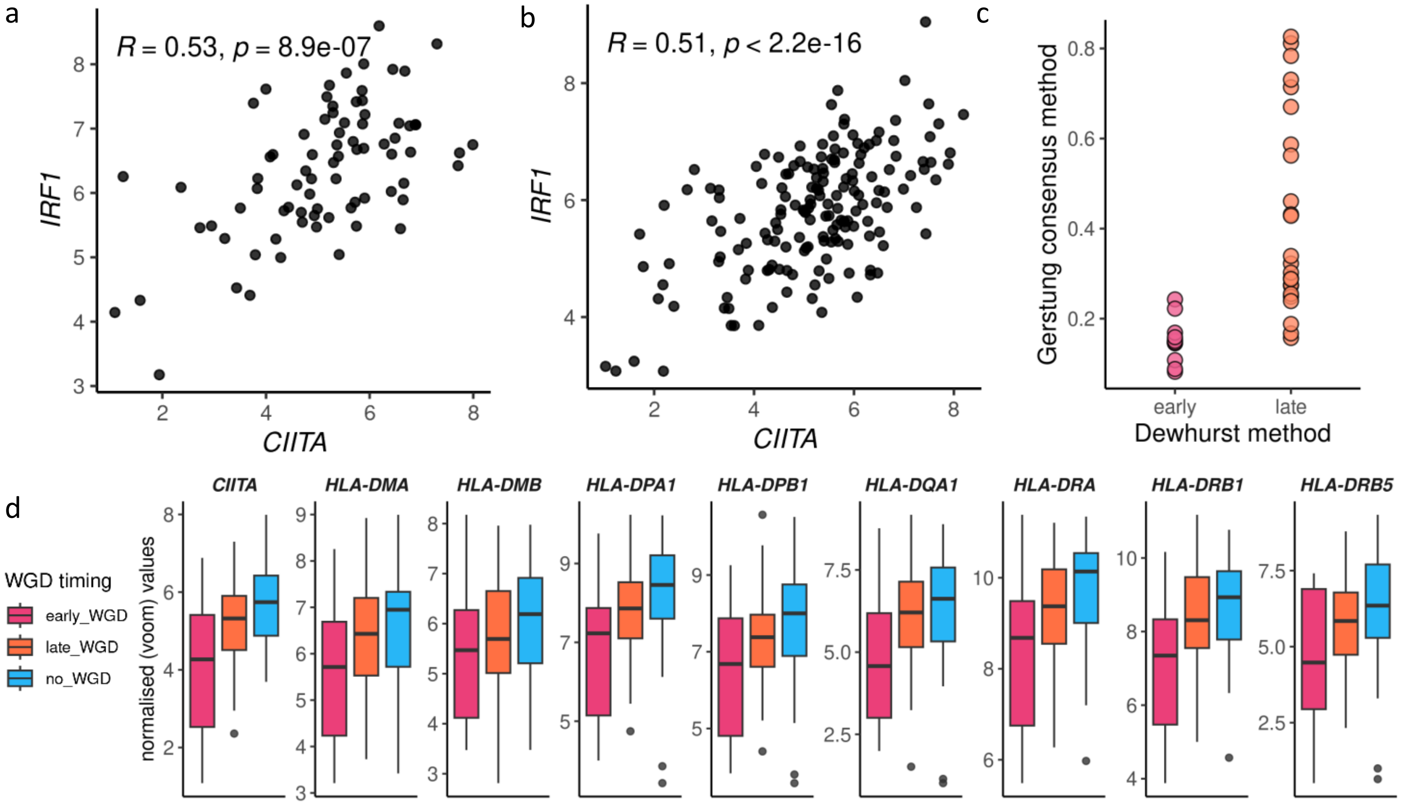

	CIITA	*HLA-DMA*	*HLA-DMB*	*HLA-DPA1*	*HLA-DPB1*	*HLA-DQA1*	*HLA-DRA*	*HLA-DRB1*	*HLA-DRB5*
p value	0.011	0.044	0.326	0.014	0.036	0.026	0.018	0.011	0.067
adj. p	0.041	0.057	0.326	0.041	0.054	0.048	0.041	0.041	0.075

e

**Characteristic**	**log(HR)**[1]	**95% CI**[1]	**p-value**
WGD_status			
early_WGD	—	—	
late_WGD	-0.54	-0.88, -0.20	0.002
no_WGD	-0.44	-0.82, -0.06	0.025
Age	0.02	0.01, 0.04	<0.001
Stage			
[Not Available]	—	—	
IB	-17	-15,446, 15,413	>0.9
IC	-0.49	-2.5, 1.5	0.6
IIA	-15	-2,606, 2,575	>0.9
IIB	-1.0	-3.4, 1.4	0.4
IIC	-1.2	-3.0, 0.61	0.2
IIIA	-0.24	-2.1, 1.6	0.8
IIIB	-0.68	-2.4, 1.0	0.4
IIIC	-0.35	-1.8, 1.1	0.6
IV	-0.24	-1.7, 1.2	0.7

[1] HR = Hazard Ratio, CI = Confidence Interval

f

**Characteristic**	**log(HR)**[1]	**95% CI**[1]	**p-value**
WGD_status			
early_WGD	—	—	
late_WGD	-0.41	-0.74, -0.09	0.012
no_WGD	-0.56	-0.92, -0.19	0.003
Age	0.00	-0.01, 0.02	0.4
Stage			
[Not Available]	—	—	
IB	-12	-3,030, 3,006	>0.9
IC	0.91	-1.4, 3.2	0.4
IIA	0.08	-2.7, 2.9	>0.9
IIB	0.16	-2.6, 2.9	>0.9
IIC	0.72	-1.4, 2.8	0.5
IIIA	0.46	-1.8, 2.7	0.7
IIIB	0.50	-1.7, 2.7	0.6
IIIC	1.3	-0.64, 3.3	0.2
IV	1.4	-0.58, 3.4	0.2

[1] HR = Hazard Ratio, CI = Confidence Interval

Supplementary Fig. 2. Bulk RNAseq analyses.

a. Correlation of expression of *CIITA* and its positive regulator *IRF1* in the ICGC cohort, Spearman's rho = ICGC 0.53, p<0.001, and b. the TCGA cohort, Spearman's rho = 0.51, p<0.001. n = 79 patient samples. c. Comparison of numerical value inferred by Gerstung et al. for relative timing of whole genome duplication (WGD; y axis) and categorical method by Dewhurst et al. (x axis) Early WGD n = 12, Late WGD n = 24. d. Boxplots of *CIITA* and MHC-II gene expression in the ICGC cohort by timing of WGD; comparisons performed with Kruskal-Wallis test. Left and right whiskers terminate at the minimum and maximum values no further than 1.5× interquartile range; center line represents median (50th percentile); left and right boundaries of box represent the first (25th percentile) and third (75th percentile) quartiles, respectively; outlying values are plotted as individual points beyond whiskers. Early WGD n = 16 patient samples, Late WGD n = 34 patient samples, No WGD = 29 patient samples e. Summary of Cox proportional hazard multivariate analysis for overall survival (OS) by WGD status. f. Summary of Cox proportional hazard multivariate analysis for progression free survival (PFS) by WGD status. Source data are provided as a Source Data file.

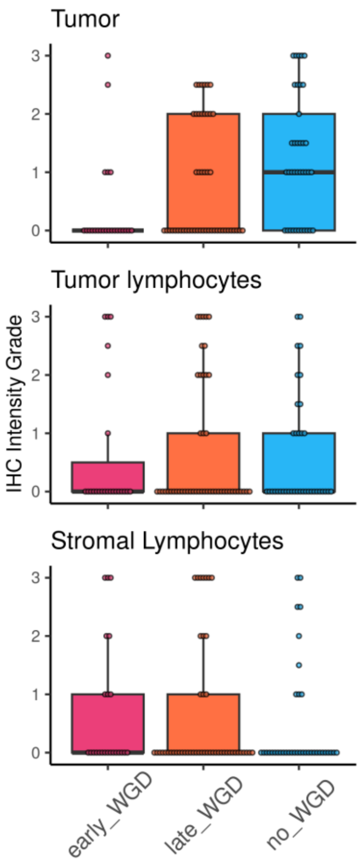

Location	Statistic	Adj. p value
Tumor cells	4.276	0.049
TILs	0.427	0.734
Stromal lymphocytes	0.311	0.734

Supplementary Fig. 3. Immunohistochemistry staining intensity results.

Boxplots overlaid with dots to show distribution of values, showing immunohistochemistry (IHC) intensity by timing of WGD and by cell type (tumor, tumor infiltrating lymphocyte (TIL) and stromal lymphocyte). Joint p values comparing IHC intensity derived from generalized linear mixed modelling on each location included in table below plots. Left and right whiskers terminate at the minimum and maximum values no further than 1.5× interquartile range; center line represents median (50th percentile); left and right boundaries of box represent the first (25th percentile) and third (75th percentile) quartiles, respectively; outlying values are plotted as individual points beyond whiskers. Early WGD n = 23 cores; Late WGD n = 49 cores; No WGD n = 37 cores. Source data are provided as a Source Data file.

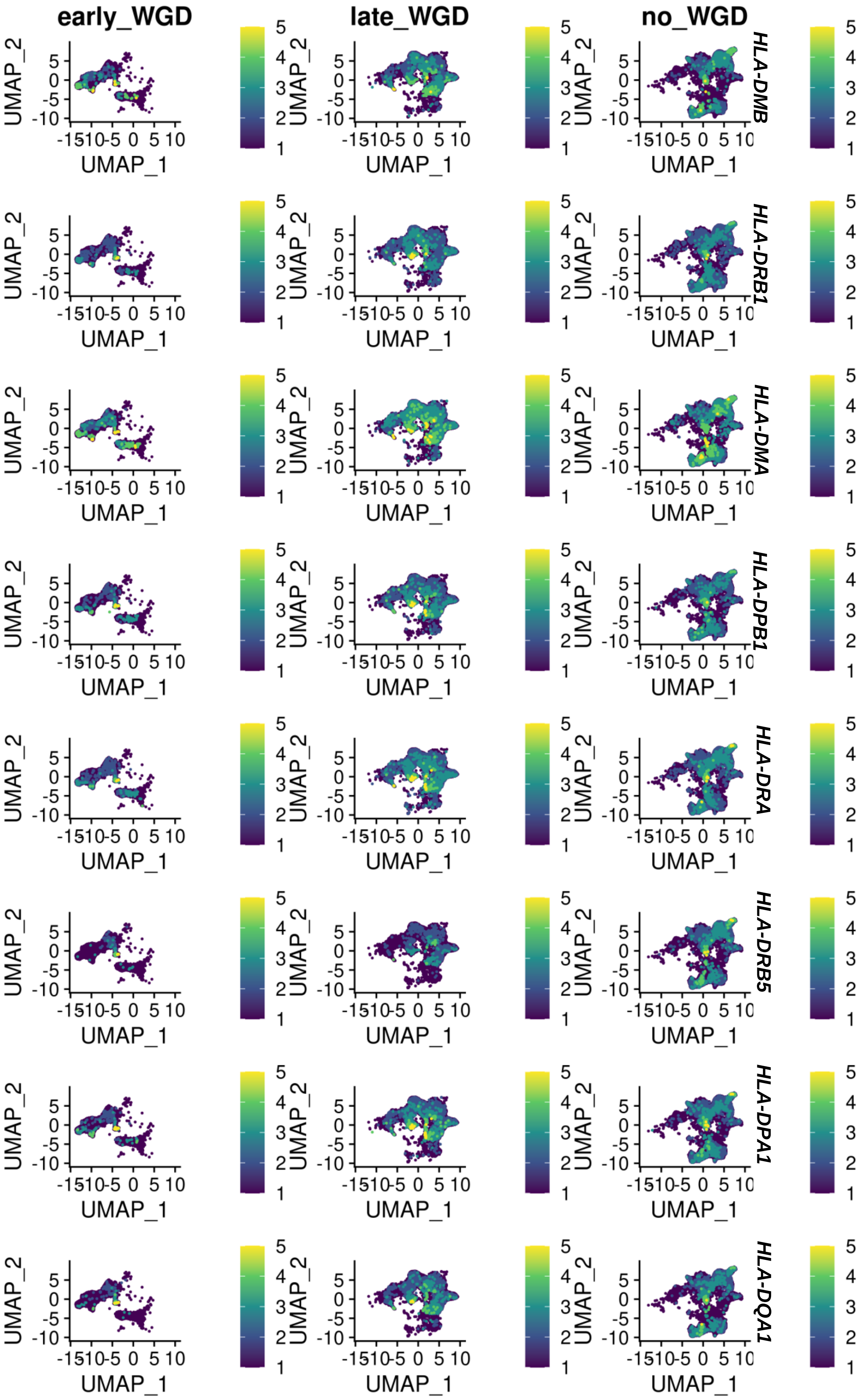

Supplementary Fig. 4. Expression of MHC-II genes in the snRNAseq data by WGD status.

Feature UMAP plots of HLA genes which were significant in the bulk RNAseq DGE analysis, showing their expression by whole genome duplication (WGD) status category. Early WGD n = 4 patients, 31,479 nuclei; Late WGD n = 5 patients, 48,685 nuclei; No WGD n = 5 patients, 60,602 nuclei.

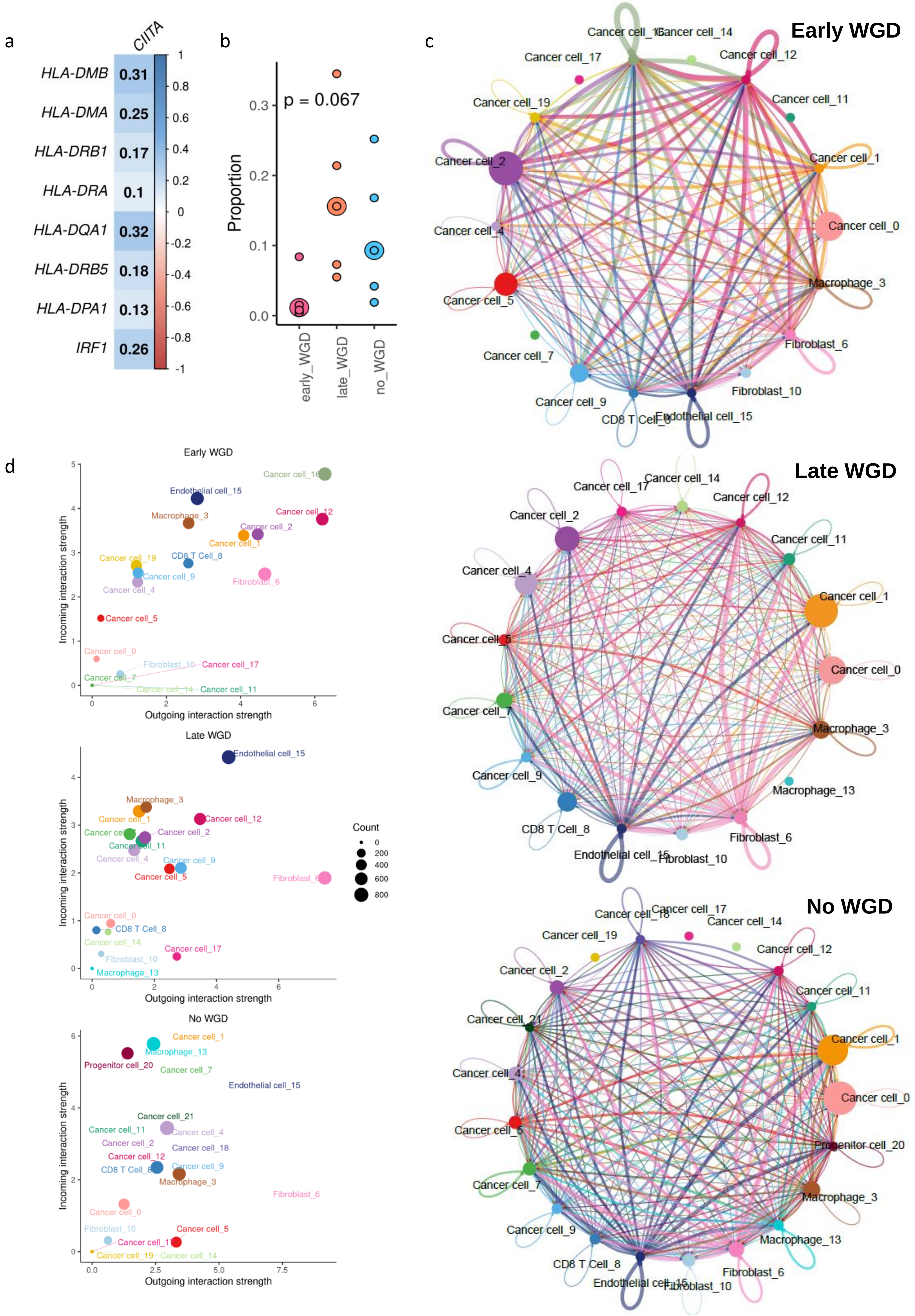
a
CIITA
HLA-DMB 0.31
HLA-DMA 0.25
HLA-DRB1 0.17
HLA-DRA 0.1
HLA-DQA1 0.32
HLA-DRB5 0.18
HLA-DPA1 0.13
IRF1 0.26
1
0.8
0.6
0.4
0.2
0
-0.2
-0.4
-0.6
-0.8
-1
b
p = 0.067
Proportion
0.0
0.1
0.2
0.3
early_WGD
late_WGD
no_WGD
c
Early WGD
Late WGD
No WGD
Cancer cell_0
Cancer cell_1
Cancer cell_2
Cancer cell_4
Cancer cell_5
Cancer cell_7
Cancer cell_9
Cancer cell_11
Cancer cell_12
Cancer cell_14
Cancer cell_16
Cancer cell_17
Cancer cell_18
Cancer cell_19
Cancer cell_21
Macrophage_3
Macrophage_13
Fibroblast_6
Fibroblast_10
CD8 T Cell_8
Endothelial cell_15
Progenitor cell_20
d
Early WGD
Late WGD
No WGD
Incoming interaction strength
Outgoing interaction strength
Count
0
200
400
600
800

Supplementary Fig. 5 MHC-II gene expression in cancer cells.

a. Correlation of *CIITA*, *IRF1* and selected MHC-II gene expression in cancer cells in snRNAseq. Scale bar indicates correlation R value. All adjusted p values <0.001, calculated with CSCORE. n = 14 patients, 140,766 cancer cell nuclei. b. Dotplot of proportion of cells which are annotated as immune cells as a fraction of total cells, per patient, by whole genome duplication (WGD) status (R = 0.067, Kruskal-Wallis test); individual patients depicted as dots, median by category depicted by larger circle. Early WGD n = 4 patients; Late WGD n = 5 patients; No WGD n = 5 patients. c. Circle network plots visualizing statistically significant interactions between cell clusters. Outer ring and circle color represents cluster identity, line weight represents interaction strength; internal color band represents the cluster of the target of the outgoing signal, the arrow points also to the target of the incoming signal. d. Bubble plots depicting the strength of ingoing and outgoing interactions with other cell subsets, divided by WGD timing category. Count represented by size of circle. Colors (also reflected in Fig.4) enable easier distinguishing between cell subsets n = 177,801 nuclei. Source data are provided as a Source Data file and Supplementary Table 10.
