# Supplementary material for: Timing of whole genome duplication is associated with tumor-specific MHC-II depletion in serous ovarian cancer
Source: Nat Commun. 2024 Jul 18;15:6069. doi: 10.1038/s41467-024-50137-y (PMC11258338; doi:10.1038/s41467-024-50137-y)
Supplement: Supplementary file 3 — Description of Additional Supplementary Files [file 41467_2024_50137_MOESM3_ESM.pdf]

Description of Additional Supplementary Files

File Name: Supplementary Data 1
Description: **FACETS purity**
FACETS purity estimation by sample for ICGC dataset

File Name: Supplementary Data 2
Description: **Differential gene expression analysis for both ICGC & TCGA cohort**
689 genes which are statistically significant in both datasets (glm). P values are adjusted for multiple comparisons

File Name: Supplementary Data 3
Description: **Differential gene expression analysis for both ICGC & TCGA cohort - without copy number log ratio as a covariate**
550 genes which are statistically significant in both datasets (glm). P values are adjusted for multiple comparisons

File Name: Supplementary Data 4
Description: **TCGA Hallmark pathway enrichment score clustering**
Normalised Hallmark pathway enrichment score clusters for the 166 TCGA samples

File Name: Supplementary Data 5
Description: **CIBERSORTx imputations - statistical comparisons**
Comparison of CIBERSORTx imputations per cell subset between WGD status categories. Kruskal-wallis test p values adjusted using Benjamini-Hochberg method.

File Name: Supplementary Data 6
Description: **CIBERSORTx imputations - raw data**
CIBERSORTx imputations for 78 samples (data with $p \geq 0.05$ excluded)

File Name: Supplementary Data 7
Description: **Characteristics of cases for snRNAseq**
Clinical characteristics of cases for snRNAseq

File Name: Supplementary Data 8
Description: **snRNAseq cell annotations**
Single nuclei RNA sequencing final cell clusters, WGD status, aneuploidy prediction, and cell annotations

File Name: Supplementary Data 9
Description: **Proportions of cell types recovered in snRNAseq**
Proportions of cell types recovered in snRNAseq by WGD status

File Name: Supplementary Data 10
Description: **CellChat ligand-receptor pairs**
Statistically significant interactions inferred by CellChat, which internally adjusts for multiple comparisons (bonferroni method). Threshold for statistical significance is $p < 0.05$.
