# Supplementary material for: Timing of whole genome duplication is associated with tumor-specific MHC-II depletion in serous ovarian cancer
Source: Nat Commun. 2024 Jul 18;15:6069. doi: 10.1038/s41467-024-50137-y (PMC11258338; doi:10.1038/s41467-024-50137-y)
Supplement: Supplementary file 5 — Reporting Summary [file 41467_2024_50137_MOESM5_ESM.pdf]

nature portfolio

Corresponding author(s): Liz Christie

Last updated by author(s): Jun 27, 2024

Reporting Summary

Nature Portfolio wishes to improve the reproducibility of the work that we publish. This form provides structure for consistency and transparency in reporting. For further information on Nature Portfolio policies, see our Editorial Policies and the Editorial Policy Checklist.

Statistics

For all statistical analyses, confirm that the following items are present in the figure legend, table legend, main text, or Methods section.

n/a	Confirmed	
☐	☒	The exact sample size (*n*) for each experimental group/condition, given as a discrete number and unit of measurement
☐	☒	A statement on whether measurements were taken from distinct samples or whether the same sample was measured repeatedly
☐	☒	The statistical test(s) used AND whether they are one- or two-sided<br>*Only common tests should be described solely by name; describe more complex techniques in the Methods section.*
☐	☒	A description of all covariates tested
☐	☒	A description of any assumptions or corrections, such as tests of normality and adjustment for multiple comparisons
☐	☒	A full description of the statistical parameters including central tendency (e.g. means) or other basic estimates (e.g. regression coefficient) AND variation (e.g. standard deviation) or associated estimates of uncertainty (e.g. confidence intervals)
☐	☒	For null hypothesis testing, the test statistic (e.g. *F*, *t*, *r*) with confidence intervals, effect sizes, degrees of freedom and *P* value noted<br>*Give P values as exact values whenever suitable.*
☒	☐	For Bayesian analysis, information on the choice of priors and Markov chain Monte Carlo settings
☒	☐	For hierarchical and complex designs, identification of the appropriate level for tests and full reporting of outcomes
☐	☒	Estimates of effect sizes (e.g. Cohen's *d*, Pearson's *r*), indicating how they were calculated

Our web collection on statistics for biologists contains articles on many of the points above.

Software and code

Policy information about availability of computer code

Data collection	The Australian Ovarian Cancer Study Group used a confidential Microsoft Access database for data collection.
Data analysis	The 10x Chromium platform was used to generate single nuclei RNAseq (snRNAseq) libraries using the 10x Genomics single cells 3` reagent kit v3.1. Libraries were sequenced on the Illumina NovaSeq6000 (paired end 150bp reads, 40,000 reads per cell).<br><br>Single nuclei RNA sequencing was aligned to the reference genome GRCh38 using CellRanger.<br><br>All post-processing analyses were conducted in Rstudio v4.1.0.<br>WGS and bulk RNAseq analyses###<br>rtracklayer_1.54.0<br>VariantAnnotation_1.38.0<br>Rsamtools_2.10.0<br>Biostrings_2.62.0<br>XVector_0.34.0<br>GenomicFeatures_1.44.2<br>AnnotationDbi_1.56.2<br>csaw_1.26.0<br>AnnotationHub_3.0.2

BiocFileCache_2.2.1
dbplyr_2.2.1
DT_0.21
SummarizedExperiment_1.24.0
Biobase_2.54.0
GenomicRanges_1.46.1
GenomeInfoDb_1.30.1
IRanges_2.28.0
S4Vectors_0.32.4
BiocGenerics_0.40.0
MatrixGenerics_1.6.0
TCGAbiolinks_2.20.0 (except 2.31.2 for methylation array data)
survminer_0.4.9
survival_3.3-1
ComplexHeatmap_2.8.0
ggpubr_0.4.0
ggrepel_0.9.1
forcats_0.5.1
ActivePathways_1.1.1
factoextra_1.0.7
patchwork_1.1.1
doParallel_1.0.17
iterators_1.0.14
foreach_1.5.2
gtools_3.9.4
purrr_0.3.4
DHARMa_0.4.5
stringr_1.5.0
mgsub_1.7.3
matrixStats_0.62.0
broom_1.0.0
tidyr_1.2.0
ggsci_2.9
edgeR_3.34.0
limma_3.50.3
ggplot2_3.4.2
dplyr_1.0.9
data.table_1.14.8
glmmTMB_1.1.3
pwr_1.3-0

snRNAseq###
future_1.33.0
glmGamPoi_1.4.0
sctransform_0.3.5
doParallel_1.0.17
iterators_1.0.14
foreach_1.5.2
DoubletFinder_2.0.3
glmmTMB_1.1.3
DHARMa_0.4.5
tidyseurat_0.6.1
ttservice_0.3.6
copykat_1.1.0
ggplot2_3.4.2
CSCORE_0.0.0.9000
ggpubr_0.4.0
emmeans_1.7.5
corrplot_0.92
harmony_0.1.0
ggtree_3.2.1
Rcpp_1.0.11
patchwork_1.1.2
forcats_1.0.0
ggsci_2.9
HGNChelper_0.8.1
SeuratObject_4.1.3
Seurat_4.1.1 for filtering, 4.3.0.1 (Open on Demand) for downstream analyses

dplyr_1.1.2
data.table_1.14.8
CellChat_1.6.1
Biobase_2.54.0
BiocGenerics_0.40.0
igraph_1.5.0
viridis_0.6.2
stats_4.1.0

No custom software was used.
Demonstrative R markdown files demonstrating analysis and code have been uploaded to synapse.org (restricted view) and are available on request to corresponding author.

For manuscripts utilizing custom algorithms or software that are central to the research but not yet described in published literature, software must be made available to editors and reviewers. We strongly encourage code deposition in a community repository (e.g. GitHub). See the Nature Portfolio guidelines for submitting code & software for further information.

Data

Policy information about availability of data

All manuscripts must include a data availability statement. This statement should provide the following information, where applicable:

- Accession codes, unique identifiers, or web links for publicly available datasets
- A description of any restrictions on data availability
- For clinical datasets or third party data, please ensure that the statement adheres to our policy

All bulk RNAseq, whole genome sequencing and methylation data used in this study has previously been published. The ICGC publicly available data (24, 73) used in this study are available in the European Genome-Phenome archive under accession code EGAD00001000877. The TCGA publicly available data (53) used in this study are available from https://portal.gdc.cancer.gov/. Due to the sensitive nature of these patient datasets, access is subject to approval from the ICGC Data Access Compliance Office (https://docs.icgc.org/download/data-access/). ICGC methylation data sets have been deposited into the Gene Expression Omnibus (GEO; https://www.ncbi.nlm.nih.gov/geo/) under accession code GSE65821, without access restrictions. ICGC gene count level transcriptomic data has been deposited into the GEO under accession code GSE209964. ABSOLUTE purity estimates and copy number signatures were downloaded directly from supplementary information (54, 55).

The snRNAseq raw data generated in this study have been deposited in the European Genome-Phenome archive under the accession code EGAD50000000364. Due to the sensitive nature of these patient datasets, the data is available under restricted access, which can be obtained by contacting DGO@petermac.org. Responses to data requests aim to be provided within two weeks. Access will be granted for appropriate research use, which are in line with the original consent provided through AOCS. Duration of data access once granted is not restricted. Processed data are available in Supplementary Tables. The remaining data are available within the Article, Supplementary Information or Source Data file.

Research involving human participants, their data, or biological material

Policy information about studies with human participants or human data. See also policy information about sex, gender (identity/presentation), and sexual orientation and race, ethnicity and racism.

Reporting on sex and gender	This study involved participants with ovarian cancer, relevant only to individuals of biologically female sex, therefore neither sex nor gender is reported.
Reporting on race, ethnicity, or other socially relevant groupings	This information was not included in this study.
Population characteristics	Fifteen individuals who had high grade serous ovarian cancer and had consented to participation in the Australian Ovarian Cancer Study. All samples were from primary diagnosis. Ages were between 36-89, and FIGO stage ranged from IB-IV
Recruitment	Fifteen individuals who had high grade serous ovarian cancer had consented to participation in the Australian Ovarian Cancer Study.
Ethics oversight	Peter MacCallum Cancer Centre Ethics Committee

Note that full information on the approval of the study protocol must also be provided in the manuscript.

Field-specific reporting

Please select the one below that is the best fit for your research. If you are not sure, read the appropriate sections before making your selection.

☒ Life sciences ☐ Behavioural & social sciences ☐ Ecological, evolutionary & environmental sciences

For a reference copy of the document with all sections, see nature.com/documents/nr-reporting-summary-flat.pdf

Life sciences study design

All studies must disclose on these points even when the disclosure is negative.

Sample size	For survival analysis, the pwr.anova.test() function from the pwr R package (v1.3-0) was used to determine sample size. For the remainder of analyses, no sample size calculations were conducted, and sample size was pragmatic.
Data exclusions	No samples were excluded from the bulk RNA sequencing<br>One sample was excluded from single nuclei RNA sequencing as sufficient nuclei could not be extracted
Replication	Analyses were performed on finite patient samples, and for much of the study, existing data, hence analyses were not replicated.
Randomization	No randomisation was undertaken.
Blinding	The pathologist who scored HLA DR + DP + DQ antibody staining had no information on the WGD status of patients. Otherwise, no blinding was performed.

Reporting for specific materials, systems and methods

We require information from authors about some types of materials, experimental systems and methods used in many studies. Here, indicate whether each material, system or method listed is relevant to your study. If you are not sure if a list item applies to your research, read the appropriate section before selecting a response.

Materials & experimental systems

n/a	Involved in the study	
☐	☒	Antibodies
☒	☐	Eukaryotic cell lines
☒	☐	Palaeontology and archaeology
☒	☐	Animals and other organisms
☐	☒	Clinical data
☒	☐	Dual use research of concern
☒	☐	Plants

Methods

n/a	Involved in the study	
☒	☐	ChIP-seq
☒	☐	Flow cytometry
☒	☐	MRI-based neuroimaging

Antibodies

Antibodies used	anti-HLA DR + DP + DQ antibody [clone CR3/43] (Abcam Cat# ab7856, lot# GR3434335-1); dilution 1:150; concentration 1.33ng/uL
Validation	Antibody was optimised on ovarian tumor tissue and non-malignant tonsil sections at a dilution of 1:150<br>https://www.abcam.com/products/primary-antibodies/hla-dr--dp--dq-antibody-cr343-ab7856.html

Clinical data

Policy information about clinical studies

All manuscripts should comply with the ICMJEguidelines for publication of clinical research and a completedCONSORT checklist must be included with all submissions.

Clinical trial registration	Not applicable - not a clinical trial
Study protocol	Not applicable - not a clinical trial
Data collection	Ambispective collection of data from clinical notes from individuals who had high grade serous ovarian cancer and had consented to participation in the Australian Ovarian Cancer Study.
Outcomes	Not applicable - not a clinical trial

Plants

Seed stocks

Report on the source of all seed stocks or other plant material used. If applicable, state the seed stock centre and catalogue number. If plant specimens were collected from the field, describe the collection location, date and sampling procedures.

Novel plant genotypes

Describe the methods by which all novel plant genotypes were produced. This includes those generated by transgenic approaches, gene editing, chemical/radiation-based mutagenesis and hybridization. For transgenic lines, describe the transformation method, the number of independent lines analyzed and the generation upon which experiments were performed. For gene-edited lines, describe the editor used, the endogenous sequence targeted for editing, the targeting guide RNA sequence (if applicable) and how the editor was applied.

Authentication

Describe any authentication procedures for each seed stock used or novel genotype generated. Describe any experiments used to assess the effect of a mutation and, where applicable, how potential secondary effects (e.g. second site T-DNA insertions, mosiacism, off-target gene editing) were examined.
